# Orthokeratology Lens Decentration with Two Designs of Corneal Refractive Therapy™ Lenses: A One-Year Prospective Study

**DOI:** 10.3390/jcm13247567

**Published:** 2024-12-12

**Authors:** Laura Batres, Cristina Arroyo-del Arroyo, Julia Bodas-Romero, Gonzalo Carracedo

**Affiliations:** 1 Ocupharm Research Group, Department of Optometry and Vision, Faculty of Optics and Optometry, Complutense University of Madrid, 28037 Madrid, Spain; lbatres@ucm.es (L.B.); crarro01@ucm.es (C.A.-d.A.); jbodas@ucm.es (J.B.-R.); 2 IOBA (Institute of Applied Ophthalmobiology), University of Valladolid, 47002 Valladolid, Spain

**Keywords:** orthokeratology, decentration, treatment zone, CRT

## Abstract

**Background/Objectives**: The objective of this study was to examine the trend of treatment zone (TZ) decentration over 12 months of orthokeratology (OK) wear using two Corneal Refractive Therapy (CRT) lens designs: standard (STD) and dual axis (DA). **Methods**: A prospective, randomized, longitudinal study was conducted at the Optometry Clinic of the Complutense University of Madrid. Subjects were randomly fitted with an STD design or DA design in one of the eyes. Refraction, uncorrected visual acuity (VA), and corneal topography were performed at baseline and after 1 night, 1 week, and 1, 3, 6, and 12 months of lens wear. Subjects requiring lens parameter adjustments or replacements after 3 months were excluded. Decentration was measured by subtracting pre-OK from post-OK tangential curvature maps at each visit, with decentration distance and corneal optical TZ being measured using MATLAB. Correlations between decentration and visual acuity (VA) were also analyzed. **Results**: A total of 30 healthy children (17 females and 13 males) with a mean age of 12.83 ± 2.42 years (range: 8–17 years) completed all the visits. No statistically significant differences (*p* > 0.05) were found between lens designs in horizontal, vertical, nor total decentration through the visits. However, for the STD design, horizontal and total decentration increased significantly at the last visit (*p* < 0.05). For the DA design, no significant differences were found over time (*p* > 0.05). No correlation was found between decentration and VA. **Conclusions**: Total decentration in both lens designs was similar throughout one year of follow-up. The standard design tended to decenter horizontally during the last 6 months, while the dual-axis design maintained consistent decentration throughout the year.

## 1. Introduction

The fitting of orthokeratology (OK) lenses has been growing in clinical practice in recent years due to rising cases of myopia. In 2023, OK lens fittings represented 1% of global contact lens adaptations, while in Spain, this percentage was notably higher, reaching 4% [1]. OK offers a non-surgical alternative for vision correction, particularly for individuals with mild to moderate myopia or for myopia management in children and adolescents. The main principle of OK involves reshaping the cornea by the hydraulic forces within the tear-lens pressure on the eye while sleeping [2].

The changes in corneal curvature induced by wearing OK lenses create an optical zone or treatment zone (TZ) dependent on the magnitude of the refractive error to be corrected or on the wearing time [3,4]. To achieve successful optical outcomes, the TZ should be centered on the cornea. The estimated displacement between the pupil center and the nominal TZ center indicates lens fitting decentration. The decentration of OK lenses is a prevalent and inevitable occurrence even when fitted optimally. The complications resulting from lens decentration lead to a reduction in contrast sensitivity, causing symptoms such as halos, glare, and blurred vision [5,6].

Although the mechanism behind the decentration of OK lenses remains unclear, some studies have suggested that it is a multifactorial phenomenon [6,7,8]. In the literature, it has been correlated with corneal astigmatism, considering different lens designs and material permeability [3,6,9]. In the study by Yang et al. [8], a significant correlation was found between the magnitude of decentration, lens diameter, and corneal toricity, where smaller lens diameters and higher corneal toricity were associated with greater lens decentration. Another study further confirmed the relationship between lens decentration and corneal toricity [7], showing that the magnitude of lens decentration in the moderate-toricity group was twice as large as that in the low-toricity group.

It has also been observed that OK lenses tend to decenter to the temporal and inferior quadrants of the cornea [6,8]. These authors suggested that corneal asymmetry might be the primary contributing factor to this phenomenon.

According to several studies, a high prevalence of astigmatism in children with myopia [10,11,12,13] has been reported, showing that myopia and astigmatism often co-occur, particularly in school-aged children. Therefore, a high proportion of corneal astigmatism patients have relative contraindications to traditional OK lens fitting because of remarkable lens decentration and poor visual quality. In response to this issue, toric or dual-axis ortho-k lenses have been developed; they have since demonstrated improved lens centration in patients with moderate to high corneal astigmatism [14,15,16,17].

Nevertheless, despite establishing a central position initially, toric lens wearers sometimes experience gradual lens decentration during follow-up visits. Diverse designs of the posterior surface may have distinct impacts on corneal reshaping. However, there is a lack of published papers measuring the decentration of two OK lens designs. Considering the impact of OK decentration on the effectiveness and satisfaction with OK lenses, this study aimed to assess the decentration of two different designs of Corneal Refractive Therapy (CRT) lenses over a year.

## 2. Materials and Methods

### 2.1. Study Design and Sample

A prospective, randomized, and longitudinal study was performed. The study was conducted in compliance with good clinical practice guidelines and the tenets of the Declaration of Helsinki and approved by the Ethics Committee of the Hospital Clinico San Carlos (Madrid, Spain). Before beginning the study, the risks and benefits of the treatment were explained, and informed consent was obtained from all subjects (and their parents or guardians).

The study had a duration of 12 months from the beginning of the treatment. The visits were categorized. Baseline measurements before initiating the treatment (PRE-OK) were performed, which provided data for assessing the feasibility of OK lens wear. Follow-up visits occurred at 1 night (1 N), 1 week (1 W), 1 month (1 M), 3 months (3 M), 6 months (6 M), and 12 months (12 M) from the beginning of OK lens wear.

Inclusion criteria were an age between 7 and 17 years, myopia less than or equal to −6.00 diopters (D), astigmatism less than or equal to −2.50 D, and best spectacle-corrected visual acuity (VA) of 0.1 logMAR or better. No previous OK lens wearers were included in this study. Exclusion criteria were a history of ocular disease and diabetes mellitus. Contact lens wearers were asked to stop wearing their contact lenses 1 week before the day of examination (PRE-OK).

### 2.2. OK Lens Fitting

All subjects were fitted with Corneal Refractive Therapy CRT™ contact lens (Paragon Vision Sciences, Gilbert, AZ, USA) in HDS 100 material (paflufocon D, Dk = 100 barrer) according to the manufacturer’s fitting guidelines. When lenses were prescribed, instructions about the wearing schedule (to wear the lenses for at least 6 h during sleep), insertion, removal, and care system were given to the subjects. For lens insertion, single-dose Lacrifresh ocu-dry artificial tears (AVIZOR, Madrid, Spain) with 0.20% of hyaluronic acid were used. For daily maintenance, the lenses were cleaned with Menicare Pure (Menicon, Nagoya, Japan) following the manufacturer’s instructions. The cleaning process was completed using the weekly disinfecting system Progent (Menicon, Nagoya, Japan). Each participant was also provided with written instructions.

The design for the initial lens for the right and left eyes of the patient was selected using a random calculation system with Microsoft Excel 2000 (Microsoft Corporation, Redmond, WA, USA). Variable 1 was assigned to the CRT™ standard design (STD), and variable 2 was assigned to the CRT™ Dual Axis design (DA). Changes in lens parameters and even in lens design, from STD to DA, were made based on fluorogram pattern and VA, especially if there was incomplete lens landing across the cornea (360°). The CRT™ lens features a sigmoidal design that replicates the curvature of the posterior surface on the anterior surface. It is divided into three zones: the base curve or optical zone (OZ), the Return Zone Depth (RZD), and the Landing Zone Angle (LZA). Furthermore, DA lenses have two meridians with different sagittal heights to achieve better centering of the lenses on toric corneas, whereas the STD design may not achieve proper centering and treatment. In both designs, the central optical zone was spherical, with a fixed diameter of 6 mm and a base curve radius (BOZR) determined by the desired refractive change.

To calculate both STD and DA lenses, the value of the flattest corneal meridian (Kf) from Oculus Pentacam (pentacam.com/int) and the sphere value obtained from subjective refraction (without distometry or spherical equivalent) were selected from both eyes. These data were entered into a calculation table provided by the manufacturer. In the eye to be fitted with the DA lens, the initial difference in the tear reservoir zone (RZD) was 25 microns.

### 2.3. Clinical Procedure

Subjects underwent a previous examination to assess ocular health and to exclude subjects with any contraindications in OK lens wear. All measurements were performed by the same experienced examiner (L.B.) at the same time each day for each subject in all visits. Measurements on the 1 N were conducted between 8:00 and 10:00 a.m. within three hours after lens removal. Follow-up measurements were performed in the evening between 7:00 and 8:00 p.m.

Refraction without cycloplegia, high and low uncorrected VA and best corrected VA, and corneal topography were performed. The topographer was calibrated before the PRE-OK measurement and at least once more during the study. Best corrected distance (4 m) VA was determined monocularly under photopic luminance conditions (85 cd/m^2^) using the ETDRS test form Chart Display VX24 (eng.visionix.ru/product/display-vx24/) with high contrast (100%) and low contrast (10%). At baseline and in the follow-up, all subjects underwent monocular testing with best correction and without correction.

### 2.4. Lens Centration Analysis

**Figure 1:**
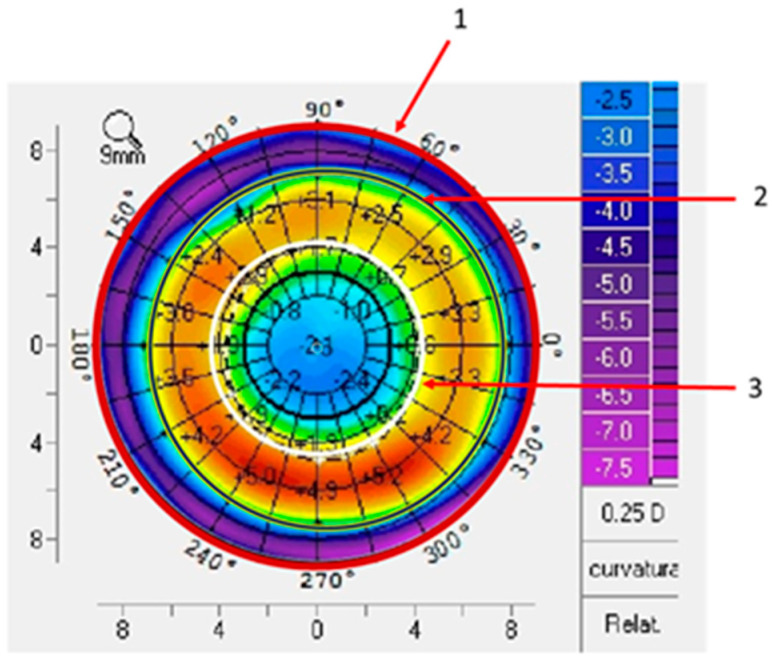
An example of a differential topographic map loaded into MATLAB. The red circle (1) represents the total area measured (delimited by marking the four outermost Cartesian points of the topography); the black circle (2) indicates the reverse curve of the lens (delimited by marking twelve points around the ring of the return zone); and the white circle (3) represents the pupil diameter (delimited by marking eight points around the pupil diameter).

For lens centration measurement, only one topography per eye was selected. A difference map was obtained by subtracting the PRE-OK lens tangential curvature map from the POST-OK lens tangential curvature map at each visit. Subjects with changes in lens parameters or lens replacement due to breakage were excluded. Three measurements were taken in each differential topography (visit—PRE-OK) for each visit and each eye, and the area to be measured was delimited by marking the four outermost Cartesian points of the topography (Figure 1, circle 1). Then, twelve points corresponding to the reverse curve of the OK lens in the differential map were drawn around the ring (Figure 1, circle 2), and eight points were drawn around the pupil diameter (Figure 1, circle 3). Based on the literature [6], the corneal optical TZ and the decentration distance of the OK lens were measured on the difference map using a MATLAB program. The TZ for each design was calculated with the comparative tangential topographic map, and it was plotted surrounding the central flattened area on which the power was zero on the difference map.

The marked points were loaded into MATLAB 24 (MathWorks, Natick, MA, USA), and analyzed by using a programming code. Another blinded investigator (J.B), different from the experienced examiner (L.B), was trained to perform the analysis. Three measurements of horizontal and vertical decentration were calculated from the comparative tangential topographic map. The formulas used to calculate the centration were the following:

- Horizontal decentration: X = the mean value of the 3 measurements obtained from the distance from the center of the topography ring to the pupillary center;
- Vertical decentration: Y = the mean value of the 3 measurements obtained from the distance from the center of the topography ring to the pupillary center;
- Total decentration: $\surd (x^{2}+y^{2})$;

$$\mathrm{Axis}:\;\frac{1}{2}*arctg\;(\frac{x}{y}).$$

For the axis calculation, the following were considered:

- If *x* and *y* > 0°, the axis was between 0 and 90°;
- If *x* < 0°, the axis was + 90°;
- If *x* > 0 and *y* < 0, the axis was + 180°.

To standardize the results and maintain the coordinate system for both eyes, the transposition along the horizontal axis of the left eye was calculated.

### 2.5. Statistical Analysis

Sample size calculations were performed with statistical software Granmo 6.0 (Institut Municipal d’Investigació Mèdica, Barcelona, Spain). The paired repeated-measures calculation tool was used, accepting an alpha risk of 0.05 and a statistical power of 80%. Statistical analysis was performed using the SPSS Statistics 23 software (IBM, Chicago, IL, USA) and Microsoft Office Excel (Microsoft Corporation, Redmond, WA, USA). Normal distribution of variables was assessed using the Shapiro–Wilk normality test. The Wilcoxon test was used to compare changes in lens decentration across study visits. The Friedman test, followed by pairwise Wilcoxon signed-rank tests with Bonferroni correction for multiple comparisons, evaluated changes in lens centration across visits for both lenses. Spearman’s rank correlation was employed to assess the relationship between decentration and uncorrected VA. Results are reported as means (standard deviations), and statistical significance was set at *p* < 0.05.

## 3. Results

### 3.1. Study Sample and OK Lens Fitting

Sixty-four healthy subjects were recruited from the Optometry Clinic of the Faculty of Optics and Optometry (University Complutense of Madrid, Madrid, Spain). However, for this study’s purposes, subjects who had undergone any changes in lens parameters after the 3 M visit or replacement due to breakage were excluded. Changes in lens parameters were considered based on the fluorogram pattern and over-refraction so that the patient achieved the best centration and VA with the lens. A lens was considered fitted if the patient slept with it on the first night. Thus, 30 subjects (13 males and 17 females) with a mean age of 12.83 ± 2.42 years were included, of whom 3 had changes in both eyes at the 1 M visit, while 3 had changes in only one eye. Additionally, at the 3 M visit, one subject had changes in both lenses, and three subjects had changes in only one lens. Regarding the total eyes fitted with the STD lens, there were no changes in 62.30% of the lenses suggested by the calculation rule. For the DA design, this percentage was 49.25%.

The numbers of STD lenses and DA lenses fitted according to the corneal astigmatism at the end of the study are presented in Table 1.

The spherical equivalent and corneal parameters of the study sample during the baseline visit are presented in Table 2.

### 3.2. Lens Centration Analysis

According to the total decentration formula, the value of total decentration and its orientation based on the visit and lens design are presented in Table 3. No significant differences were found between OK designs in total decentration at each visit.

**Figure 2:**
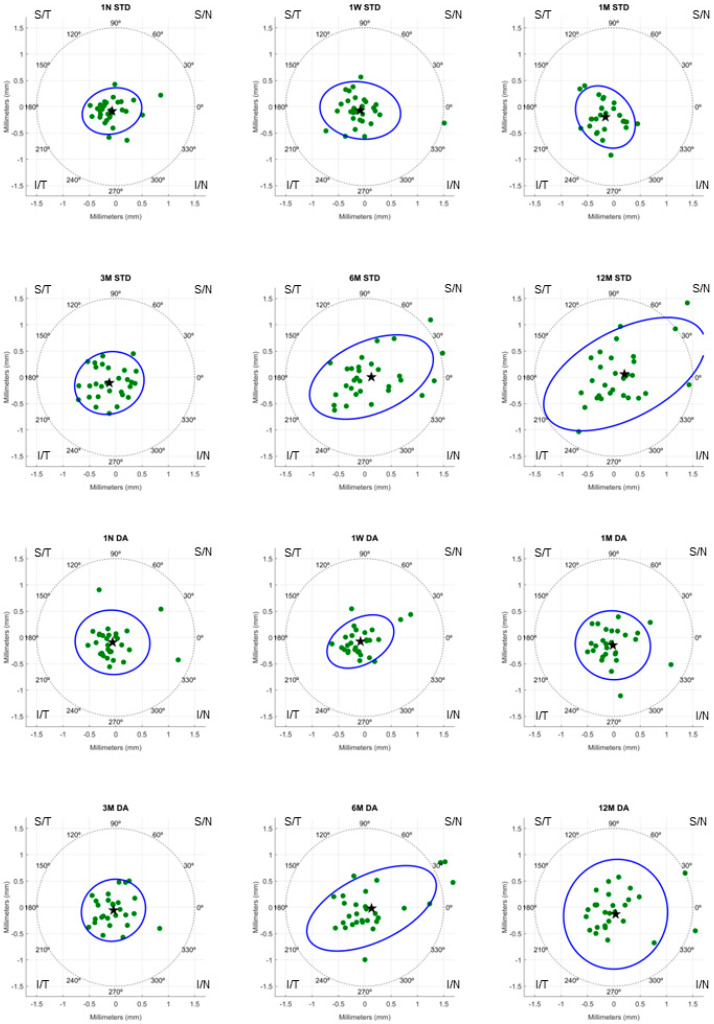
A polar coordinate representation of lens decentration throughout 12 months of orthokeratology lens wear for each group. The green dots represent the value of total decentration for each subject. The black star represents the mean value of total decentration. The blue ellipse represents the statistical dispersion of the decentration data over time, and its orientation and shape highlight the predominant direction and extent of decentration. Abbreviations: STD, standard; DA, dual axis; N, night; W, week; M, month; S, superior; T, temporal; N, nasal; I, inferior.

Figure 2 represents the decentration of STD and DA lens designs on a polar coordinate map. Each point represents the position of the vector (x, y) obtained from the corneal topography map. According to Figure 2 and Table 3, the mean total decentration of both lens designs was inferotemporal.

**Figure 3:**
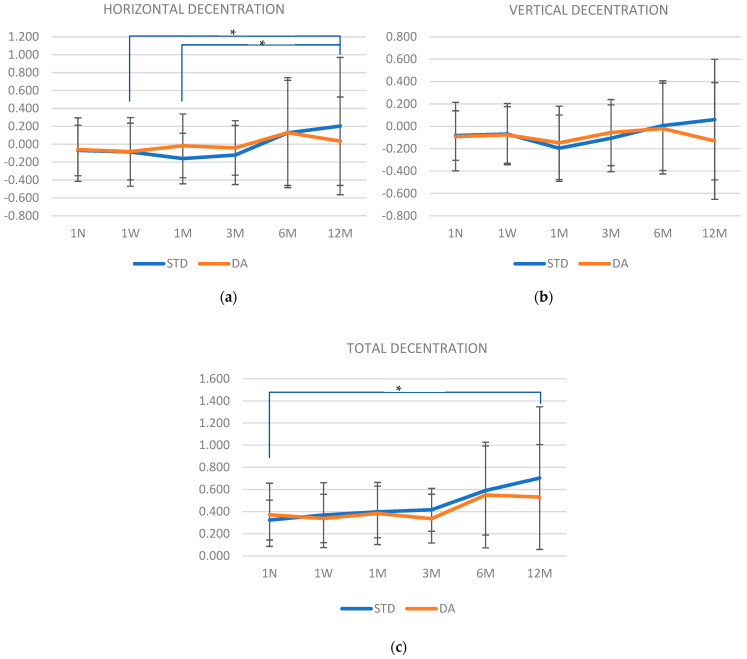
Horizontal, vertical, and total decentration amounts (denoted as (**a**–**c**), respectively) for both designs at each study visit for 30 subjects. Horizontal blue lines mark changes in decentration over the time in STD design. Abbreviations: STD, standard; DA, dual axis; N, night; W, week; M, month. * *p* < 0.05.

Figure 3a–c shows the amount of horizontal, vertical, and total decentration for both designs at each visit. No statistically significant differences (*p* > 0.05) were found between lenses in horizontal, vertical, nor total decentration through the visits. However, in Figure 3a, it can be observed that the horizontal decentration of the STD design increased significantly in the last visit (12 M) compared with the 1 W and 1 M visits in the study (*p* = 0.017 and *p* = 0.01, respectively). In addition, the total decentration (Figure 3c) for the STD design was significantly higher in the 12 M visit compared with the 1 N visit (*p* = 0.014). However, no differences were found in vertical decentration for the STD design. Meanwhile, decentration of the DA design was constant through all the visits (*p* > 0.05).

Uncorrected VA during the visits for both designs is presented in Table 4. No differences were found in VA between lenses. In addition, a Spearman correlation did not show any significant correlation between decentration and uncorrected VA for both designs in all visits (*p* < 0.3; *p* > 0.05).

## 4. Discussion

The decentration of OK lenses and their influential factors has long been a concern in clinical practice. It cannot be completely avoided as it is the main cause of about 50% of complications [18]. It has been shown that nearly 0.5% of all clinical OK wearers experiment OK decentration [19]. The first month of OK treatment is critical for corneal reshaping, although achieving perfect centration may be challenging due to variations in corneal parameters and lens designs [20]. Recent studies indicate that both the size of the TZ and decentration increase significantly during this period, gradually stabilizing thereafter [21,22]. Then, the TZ’s decentration showed a smooth fluctuation up to 6 M and a significant increase from 6 to 12 months [21]. For this reason, changes in lens parameters during the first 3 months were included in the analysis of this study as it is thereafter when changes in lens decentration begin to increase.

There are different methods for fitting OK lenses, ranging from empirical calculation and the use of a lens set to specific software provided by the manufacturer. For instance, in cases of high astigmatism, specific software is often used to assist in the lens fitting. For this study, the success rate with the initial lens calculated using the calculation algorithm was moderate for both designs, and 62.30% of subjects were fitted with the first lens suggested by the calculation rule. A similar percentage was found by Mika et al., who had about 60% success in fitting with the first empirically calculated lens [23]. The fitting of the DA design allows for adjustment in the periphery, improving molding when there are differences in the corneal curvature (or elevation) of the principal meridians. Adjustment using the fluorogram allows the lens design to be modified from a spherical to toric design in cases where the lens does not properly land around the 360° corneal surface or remains off-center. González-Méijome and Villa-Collar [24] analyzed the success rate of calculating the first STD lens provided by the manufacturer’s nomogram. In 92% of the cases fitted, the final prescribed lens was achieved by changing two parameters or less, with the RZD value being the parameter requiring the most changes.

Recently, Li et al. [25] published results from evaluating centration during OK lens wear with a toric periphery compared to lenses with a spherical design. In that study, the magnitude of the off-centered TZ was significantly smaller when the patient was fitted with a toric design, concluding that this design improves the success of fitting, especially in cases with high elevation differences between meridians at 8 mm. Toric lens designs, with a toric optical zone but a periphery with different curvatures or sagittal differences, are fitted to ensure complete lens support and centration. Additionally, several authors have demonstrated their effectiveness in reducing total refractive error, corneal astigmatism, and refractive astigmatism [14,26].

In 1999, Tsai and Lin [27] established the classification for corneal refractive surgery, defining mild decentration as less than 0.5 mm, moderate decentration between 0.5 and 1.0 mm, and severe decentration as greater than 1.0 mm. This classification subsequently formed the foundation for the classification of TZ decentration in OK fitting. According to this classification, throughout the 12-month period of lens wear, both designs experienced a similar mild temporal-inferior decentration of less than 0.5 mm. The spherical design exhibited slightly greater horizontal decentration, which increased at the 12-month visit. This design also showed a progressive increase in the total decentration compared to one night of wear. However, the toric design was stable during all study visits. The toric design of the midperipheral zone could make toric lenses more resistant to the gradual flattening of the central corneal curvature. Previous studies reported a similar inferotemporal decentration [28], which was most commonly observed in patients with astigmatism wearing spherical lenses [7,29]. According to Chen et al., it was observed that temporal decentration, inferior decentration, and inferotemporal decentration accounted for 84.9%, 58.5%, and 49.1% of all fitted OK lenses, respectively [19]. Temporal decentration resulted from the cornea’s steeper temporal side compared to the nasal side, while vertical decentration was presumed to be a composite result of eyelid tension, lens design, and fitting technology [6,8]. Therefore, in ten eyes with corneal astigmatism less than 1 D, a toric design was fitted even though it is not typically indicated as the first choice. Fitting a design with low toricity in the periphery resulted in more stable centration from the beginning of treatment and consequently improved visual quality in children.

There are few studies evaluating TZ decentration over a one-year follow-up period. In the most recent study by Li et al. [21], it was found that after 12 months of OK wear, 36.17% of participants experienced mild decentration, 53.19% had moderate decentration, and 10.64% had severe decentration. The mean decentration distance of the TZ in this study did not achieve moderate or severe decentrations and was smaller than that reported in other clinical studies [21,30]. This discrepancy may be attributed to the use of DA designs fitted even in patients with low astigmatism.

Although decentration in OK lenses may be beneficial in controlling the progression of myopia, this decentration can also have a negative influence in uncorrected VA, increasing glare or ghosting [18]. However, in this study, no correlations were found between decentration and uncorrected VA. Moreover, uncorrected VA did not show a difference between OK lens designs among visits. This could be explained due all the decentration being considered mild.

The current study did not explore eyelid force as a potential factor affecting the alignment between the lens and the corneal surface. When fitting OK lenses, numerous factors, such as corneal parameters, lens design, and eyelid forces, should be considered. Further research is necessary to examine how the interplay of these factors could impact OK lens fitting. Another limitation of the present study is the reduced sample size; to ensure a proper measurement, only subjects who had not undergone any changes in lens parameters after lens fitting or replacement due to breakage were included.

## 5. Conclusions

In conclusion, total decentration in both lens designs, STD and DA, were similar after a year of follow-up. The spheric design tended to decenter horizontally during the first 6 months, while the toric design achieved the maximum vertical decentration at the end of the year.

## Figures and Tables

**Table 1:** The relation between corneal astigmatism and the selected lens design.

Corneal Astigmatism (CA)	Total (n = 30 (60 Eyes))	CRT™ STD	CRT™ DA
CA ≤ 1.00 D	15	5	10
1.00 D < CA ≤ 2.00 D	13	4	9
2.00 D < CA ≤ 3.00 D	2	1	1

Abbreviations: CA, corneal astigmatism; D, diopter; STD, standard; DA, dual axis.

**Table 2:** Spherical equivalent and corneal parameters of study sample at baseline visit.

Variable	STD Group	DA Group	*p*-Value
Spherical Equivalent (D)	−2.78 ± 1.21	−2.68 ± 1.25	0.778
K(f) mm	7.89 ± 0.19	7.90 ± 0.18	0.990
K(s) mm	7.76 ± 0.19	7.74 ± 0.19	0.735
CA (D)	−0.70 ± 0.43	−0.86 ± 0.44	0.586
Corneal Eccentricity	0.53 ± 0.14	0.52 ± 0.13	0.422

Abbreviations: D: diopter; K: keratometry; f: flat; s: steep; STD: standard; DA: dual axis. Results are presented as means (standard deviation).

**Table 3:** Mean and orientation of decentration vector of each lens design at every visit.

Visit	STD		DA		*p*-Value
	Magnitude	Angle	Magnitude	Angle	
1 N	0.32 ± 0.18	193.90	0.37 ± 0.29	203.44	0.50
1 W	0.37 ± 0.29	204.40	0.34 ± 0.22	203.65	0.75
1 M	0.40 ± 0.23	226.18	0.38 ± 0.28	193.00	0.72
3 M	0.42 ± 0.19	214.36	0.34 ± 0.22	196.66	0.25
6 M	0.59 ± 0.40	183.63	0.55 ± 0.48	192.88	0.24
12 M	0.70 ± 0.64	173.50	0.53 ± 0.47	180.95	0.12

Abbreviations: STD, standard; DA, dual axis; N, night; W, week; M, month. Wilcoxon test.

**Table 4:** Uncorrected high-contrast visual acuity (measured in LogMAR) of each lens design in every visit.

Visit	STD	DA	*p*-Value
1 N	0.36 ± 0.28	0.35 ± 0.34	0.89
1 W	0.02 ± 0.20	0.01 ± 0.15	0.78
1 M	0.00 ± 0.13	−0.02 ± 0.11	0.70
3 M	−0.01 ± 0.13	−0.06 ± 0.11	0.10
6 M	−0.04 ± 0.09	−0.03 ± 0.15	0.77
12 M	−0.03 ± 0.12	−0.03 ± 0.12	0.77

Abbreviations: STD, standard; DA, dual axis; N, night; W, week; M, month. Wilcoxon test.

## Data Availability

Part of the results of this study can be found at https://produccioncientifica.ucm.es/documentos/60ee71d0b942fb22f714b537 (accessed on 15 July 2024).
